# Impact of risk factors in craniofacial mucormycosis

**DOI:** 10.4317/medoral.26789

**Published:** 2025-01-26

**Authors:** Josefina Alejandra Morales-Del Angel, Andrea Sarahí Guerra-Garza, Jorge Eduardo Juárez-Silva, Silvia Merari Macias-Alfaro, Baltazar González-Andrade, Marco Antonio Sánchez-Corella, José Luis Treviño-González

**Affiliations:** 1 Otolaryngology Head and Neck Surgery Division, “Hospital Universitario Dr. José Eleuterio González”, Monterrey, México; 2 ORCID: 0000-0002-1739-4080. MSc. Medical Specialist in Otolaryngology Head and Neck Surgery; 3 ORCID: 0009-0002-0097-5062. MD. Medical Specialist in Otolaryngology Head and Neck Surgery; 4 ORCID: 0000-0001-7766-2369. PhD. Medical Specialist in Otolaryngology Head and Neck Surgery. Head of Otolaryngology Head and Neck Surgery Division, “Hospital Universitario Dr. José Eleuterio González”, Monterrey, México

## Abstract

**Background:**

Craniofacial mucormycosis is a highly lethal infectious disease. This study aims to assess and analyze multiple variables, including clinical, socioeconomic, and biochemical markers, to identify and examine risk factors for mortality associated with this mycotic infection.

**Material and Methods:**

A retrospective analysis was conducted on 38 patients who sought medical attention at the Otolaryngology and Head and Neck Surgery Division of a tertiary-level hospital in Monterrey, Mexico. A broad range of variables was analyzed: clinical features, including the extent of mucormycosis infection; socioeconomic factors such as monthly income, marital status, geographical residence, educational level, and insurance status; as well as biochemical markers, including glucose levels, lactate dehydrogenase (LDH), C-reactive protein (CRP), erythrocyte sedimentation rate (ESR), and immune cell counts, specifically neutrophils (NEU) and lymphocytes (LYM). Statistical analysis was conducted using SPSS v26. Risk factors for mortality were evaluated using Cox regression. Overall survival (OS) was assessed with the Kaplan-Meier method. The Fisher's exact test and the Chi-square test were used for categorical variables. For median comparisons, the Student’s t-test and Mann-Whitney U test were applied; with normality assessed using the Shapiro-Wilk test. A *p-value* <0.05 was considered statistically significant.

**Results:**

Mucormycosis was associated with higher mortality in men (*p*=0.032). The disease primarily affected the paranasal sinuses (*p*=0.021) and was associated with increased mortality when involving the orbit (*p*=0.035). Additionally, compromised lymphocyte counts (LYM) (*p*=0.034) and lower educational levels (*p*=0.009) were associated with higher mortality. Individuals residing in rural areas also exhibited an elevated risk of mortality (*p* =0.023).

**Conclusions:**

Prevention strategies should focus on high-risk groups to reduce the mortality rate of craniofacial mucormycosis, particularly targeting men and individuals residing in rural areas. Special emphasis should be placed on those without education or health insurance. Early diagnosis and appropriate management are crucial for improving outcomes.

** Key words:**Mucormycosis, socioeconomic, mortality, rural residency.

## Introduction

**Figure 1:**
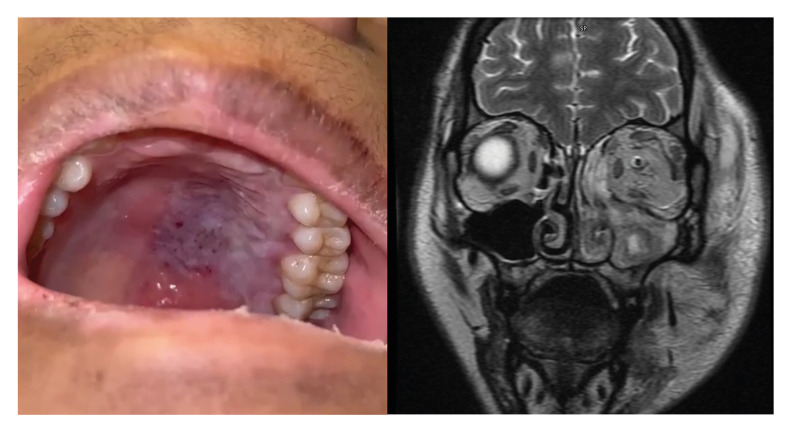
(Left) Purplish mucous lesion of left hemipalate with necrosis; (Right) Coronal section of MRI demonstrating involvement of mucormycosis infection of left ethmoid sinus, lateral wall of nose, maxillary sinus, floor and medial wall of the left orbit.

**Figure 2:**
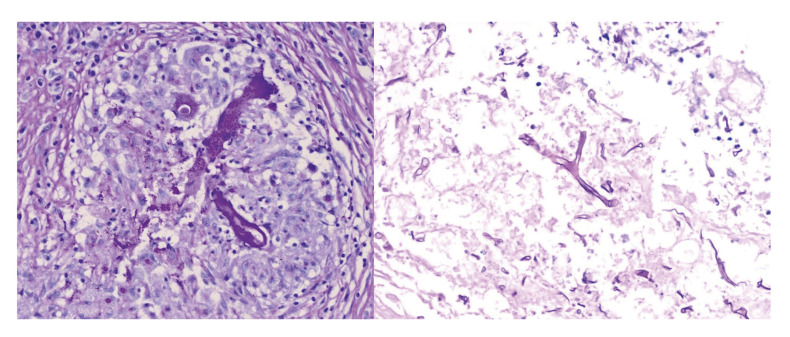
(Left) Histopathological image showing lymphocytes and epithelioid cells conforming a granuloma with a non-septate hypha in its center. PAS 40x. (Right) Necrotic tissue associated to a non-septate, irregular, thick hyphae. PAS 40x.

Mucormycosis encompasses a broad spectrum of opportunistic infections primarily characterized by angioinvasion, leading to rapid and extensive tissue necrosis (Fig. 1), which may result in significant mortality. Mortality rates are alarmingly high, with only 40% of infected individuals surviving three months after being diagnosed (1). This infection is primarily caused by fungi from the genera Rhizopus spp. and Mucor spp., which are the most prevalent (2) (Fig. 2). The virulence factors of these microorganisms and the host's immune response are crucial determinants of prognosis, with the condition predominantly affecting immunocompromised individuals (3). Although less common, immunocompetent individuals can also be affected. Globally, the incidence of mucormycosis ranges from 0.005 to 1.7 cases per million people, with an estimated incidence of 0.12 per 100,000 inhabitants in Mexico (4).

Prompt diagnosis and accurate treatment are essential to improving patient survival. Mucormycosis can present as a cutaneous infection or as more severe forms, such as rhino-orbital-cerebral mucormycosis (ROCM), which is the most common clinical presentation (5). Other forms of the disease include sino-pulmonary, gastrointestinal, and disseminated infections.

This study aims to describe and analyze the clinical, socioeconomical and biochemical markers of Hispanic patients with ROCM at a tertiary - level hospital. The main goal encompasses identifying risk factors for mortality and to raise awareness and encourage both medical and non-medical community to implement preventive measures in high - risk population in order to improve survival rates.

Materials and Methods

A retrospective study including 38 hospitalized patients who were diagnosed with mucormycosis by the Otolaryngology and Head and Neck Surgery Division from 2018 to 2023 at “Hospital Universitario Dr. José Eleuterio González” in the northeast of México was performed.

Registered patients had different comorbidities such as diabetes mellitus, high blood pressure, hematological diseases, or were otherwise immunocompetent. Diagnosis was confirmed through histopathological examination.

Demographic data, including age, sex, personal medical history (e.g., Charlson comorbidity index), treatment, and socioeconomic factors such as monthly income (USD), marital status, and residency (categorized as rural or urban) were collected. Educational level and medical insurance status were dichotomized for Cox regression analysis. Blood counts, including median glucose levels, lactate dehydrogenase (LDH), C-reactive protein (CRP), erythrocyte sedimentation rate (ESR), and immune cell counts (white blood cells [WBC] and lymphocytes [LYM]), were recorded. Additionally, disease characteristics of rhino-orbital-cerebral mucormycosis (ROCM), such as disease extension, were analyzed to determine risk factors and prognosis for mortality.

Statistical analysis was performed using SPSS v26 (IBM SPSS Statistics, IBM Corp., Armonk, NY). Cox regression was used to study risk factors for mortality, while Kaplan-Meier methods assessed overall survival. Fisher’s exact test or Chi-square test was used for categorical variables, and T-student test and Mann-Whitney U test were employed for median comparisons, with normality assessed by the Shapiro-Wilk test. A *p-value* <0.05 was considered statistically significant. The study was conducted in compliance with STROBE reporting guidelines and received approval from the local ethics committee.

## Results

- Overall clinical characteristics

The median age of the evaluated patients was 49 years, with a range from 5 to 78 years. 76.3% of them were adults, and 63.2% of the entire cohort were male. The most prevalent comorbidity was Diabetes Mellitus type 2 (DMT2), present in 60.5% of the patients, with a median of disease duration of 13 years, ranging from 5 to 20. Only 28.9 of the patients presented hematological diseases. Further baseline characteristics are descripted in Table 1.

ROCM primarily involved the paranasal sinuses (31.6%) (*p*=0.021). Functional Endoscopic Sinus Surgery (FESS) was the treatment of choice in 60.5% of cases; additionally, all patients received antifungal therapy with amphotericin B (dose adjusted based on age, comorbidities, and the severity of the disease).

The median time from symptom onset to diagnosis was 4 weeks, ranging from 1 to 39. The overall survival (OS), defined as the time from either diagnosis or treatment initiation until death from any cause, was 30.53 weeks (CI 12.24 - 48.81). A total of 12 patients (31.5%) died as a result of mucormycosis infection. Mortality was predominantly observed in men (26.3%, *n*=10) (*p*=0.032), patients with DMT2 (23.6%) (*p*=0.498), and those with orbital involvement (*p*=0.035).

- Socioeconomic features

Addressing socioeconomic aspects, 60.5% of the cohort patients were married and 68.4% of them lived within an urban residency. 73.5% had either no formal education or only basic education. 52.6% lacked medical insurance. Further information can be found in Table 2.

Among deceased patients, educational level was found to significantly impact mortality (*p*=0.009) (Table 2). Univariate analysis indicated that rural residency was associated with a sevenfold increase in mortality risk due to mucormycosis (*p*=0.023). Patients from rural areas exhibited noTable socioeconomic disparities compared to their urban counterparts, including differences in education (*p*=0.005) and insurance status (*p*<0.001).

- Biochemical markers

Laboratory tests revealed altered levels of several parameters. Most patients (60.5%) had elevated glucose levels, with a median of 189 mg/dL (range: 111.25 - 340.25 mg/dL). Inflammatory markers such as the Erythrocyte Sedimentation Rate (ESR) and C-reactive protein (CRP) were also elevated (see Table 2). In deceased patients, CRP levels were significantly higher, with a median of 21.17 mg/dL (range: 6.7 - 44.1 mg/dL) (*p*=0.003).

## Discussion

Mucormycosis is a severe and life-threatening infectious disease with a high mortality rate, ranging from 40% to 80% (6). Recognizing mortality predictors is essential for improving patient care and establishing effective preventive measures for high-risk populations. Research on this topic has been conducted in countries such as Turkey, Pakistan, Iran, and India, primarily focusing on Middle Eastern and South Asian populations (7-10). To our knowledge, this is the first retrospective cohort study addressing the Hispanic population. This gap emphasizes the need for further research into mortality predictors to improve patient-centered outcomes.

In this study, male patients were predominantly affected (63.2%) and exhibited a poorer prognosis (26.3%). Literature reports indicate a 55% mortality rate among men (11). Numerous studies from South Asia and the Middle East have also reported a higher prevalence of this disease among male patients, with low survival outcomes (12-14).

Diabetes mellitus was the most prevalent underlying condition in this retrospective cohort population (60.5%), consistent with findings from an Indian study reporting a prevalence of 97% (12). This comorbidity may facilitate the local dissemination of the infection due to microvascular complications in the sensitive structures of the sinuses (11). Complications associated with DMT2, such as ketoacidosis, are linked to increased mortality in individuals diagnosed with mucormycosis (8). The elevated free iron levels resulting from ketoacidosis offers an ideal environment for fungal proliferation (15).

Mucormycosis predominantly affects the paranasal sinuses. Nair *et al*. reported that 94.2% of patients with rhino-orbital-cerebral mucormycosis (ROCM) had frontal sinus involvement (12). In our study, the paranasal sinuses were the most frequently affected region (31.6%), with no intracranial involvement observed. Patients with orbital involvement experienced a mortality rate of 66.5%. In comparison, a retrospective cohort study conducted by Harun *et al*. reported a fatality rate of 46.1% (16). According to the global guidelines from the European Confederation of Medical Mycology, the mortality rate for central nervous system involvement is 80%, whereas the mortality rate for isolated paranasal sinus infections remains unspecified but is generally lower (6).

The socioeconomic environment significantly impacts the accessibility of medical services, thereby influencing disease prognosis. For patients residing in rural areas, a sevenfold increased risk of mortality was observed. Poswal *et al*. indicated that rural populations have a higher prevalence of mucormycosis, which may be attributed to occupations involving dust or soil, such as farming or labor (17). Our findings revealed that low educational attainment and lack of medical insurance contributed to higher mortality rates, with only 25% of deceased rural patients having insurance.

The DEFEAT Mucor study identified laboratory abnormalities such as neutropenia, elevated serum iron or ferritin levels, and malignancies, which are associated with mortality rates of 100%, 80%, 80%, and 80%, respectively (18). Mojtahedi *et al*. found that lymphopenia was the most common abnormal laboratory finding in patients with craniofacial mucormycosis (19). In this study, elevated levels of ESR and CRP were observed; however, only CRP was statistically significant (*p* < 0.05), consistent with findings previously reported by Cho *et al*. in Asian patients (20).

This study has several limitations, the foremost being the small and heterogeneous sample of patients. The population varied widely, encompassing individuals as young as 5 years with malignancies to those up to 60 years old with elevated glucose levels. Variations in treatment among patients could impact survival outcomes. Despite these limitations, the findings should be interpreted in the context of the Hispanic population studied.

To reduce mortality rates associated with craniofacial mucormycosis in the Hispanic population, prevention strategies should focus on high-risk groups, particularly men and those residing in rural areas. Special attention should be given to individuals without formal education or health insurance, with the goal of facilitating early diagnosis and timely medical-surgical intervention. Given the distinct socioeconomic and demographic characteristics of this population, further research is essential to identify specific mortality predictors and develop effective intervention strategies.

## Figures and Tables

**Table 1:** Baseline characteristics of 38 patients with mucormycosis and P differences for mortality.

Baseline characteristics		n (%)	*P value*
Age, median (range)		49 years (5 - 74)	-
Sex	Men	24 (63.2%)	0.032°
	Women	14 (36.8%)	
Recent history of COVID-19	Present	7 (18.4%)	0.676°
	Absent	31 (81.6%)	
Comorbidities	None	3 (7.9%)	0.001°
	Diabetes mellitus	23 (60.5%)	
	Arterial hypertension and DM	13 (34.2%)	
	Hematological diseases	11 (28.9%)	
	Chronic kidney disease	5 (13.2%)	
	Overweight/Obesity	7 (18.4%)	
Alcohol history	Yes	7 (18.4%)	0.767°
	No	18 (47.4%)	
	Unknown	13 (34.2%)	
Charlson index		2 (0 to 5)	0.004°

Diabetes mellitus (DM); Fisher or Chi-square test °.

**Table 2:** Socioeconomic and biochemical markers of 38 patients with mucormycosis and P differences for mortality.

Baseline characteristics		n (%)	*P value*
Socioeconomic features	Monthly Income (USD)	243.7 (111.7 to 314.8)	-
Marital status	Married	23 (60.5%)	0.761°
	Single	13 (34.2%)	
	Divorced	2 (5.3%)	
Residence	Rural	9 (23.7%)	0.063°
	Urban	26 (68.4%)	
	Unknown	3 (7.9%)	
Education	Unschooled and Basic	29 (76.5%)	0.009°
	Middle and Higher	9 (23.5%)	
Medical Insurance	Present	12 (31.6% )	0.204°
	Absent	20 (52.6 %)	
	Unknown	6 (15.8%)	
Biochemical markers	Glucose, mg/dL	189 (111.25 to 340.25)	0.096"
	LDH, IU/L	162 (139.25 to 244)	0.260"
	WBC, x10^9^/L	9.59 (0.61 to 15.77)	0.286"
	CRP, mg/dL	7.7 (7.7 to 17.9)	0.003"
	ESR, mm	34 (0 to 50)	0.279"

United States Dollar (USD); Lactate dehydrogenase (LDH); White Blood Cells (WBC); C-Reactive Protein (CRP); Fisher or Chi-square test°, Mann-Whitney U test".
